# Effect of Surface-Modified Mica in Hybrid Filler Systems on the Curing and Mechanical Behavior of Ethylene–Propylene–Diene Monomer (EPDM)/Butadiene Rubber (BR) Blend

**DOI:** 10.3390/polym17162250

**Published:** 2025-08-20

**Authors:** Won-Young Jung, Seong-Woo Cho, Keon-Soo Jang

**Affiliations:** Department of Materials Science and Engineering, School of Chemical and Materials Engineering, The University of Suwon, Hwaseong 18323, Gyeonggi-do, Republic of Korea

**Keywords:** rubber, elastomer, composite, butadiene rubber (BR), polybutadiene (PB), ethylene propylene diene monomer (EPDM), carbon black, mica

## Abstract

This study investigates the influence of hybrid filler systems comprising carbon black (CB), mica, and surface-modified mica (SM) on the properties of ethylene–propylene–diene monomer (EPDM)/butadiene rubber (PB) composites. To reduce the environmental issues associated with CB, mica was incorporated as a partial substitute, and its compatibility with the rubber matrix was enhanced through surface modification using ureidopropyltrimethoxysilane (URE). The composites with hybrid filler systems and surface modification were evaluated in terms of curing behavior, crosslink density, mechanical and elastic properties, and dynamic viscoelasticity. Rheological analysis revealed that high mica loadings delayed vulcanization due to reduced thermal conductivity and accelerator adsorption, whereas SM composites maintained comparable curing performance. Swelling tests showed a reduction in crosslink density with increased unmodified mica content, while SM-filled samples improved the network density, confirming enhanced interfacial interaction. Mechanical testing demonstrated that the rubber compounds containing SM exhibited average improvements of 17% in tensile strength and 20% in toughness. In particular, the CB20/SM10 formulation achieved a well-balanced enhancement in tensile strength, elongation at break, and toughness, surpassing the performance of the CB-only system. Furthermore, rebound resilience and Tan δ analyses showed that low SM content reduced energy dissipation and improved elasticity, whereas excessive filler loadings led to increased hysteresis. The compression set results supported the thermal stability and recovery capacity of the SM-containing systems. Overall, the results demonstrated that the hybrid filler system incorporating URE-modified mica significantly enhanced filler dispersion and rubber–filler interaction, offering a sustainable and high-performance solution for elastomer composite applications.

## 1. Introduction

Rubber composites filled with reinforcing agents are widely utilized across automotive, industrial, and consumer sectors due to their improved mechanical, thermal, and dynamic properties [1,2]. Among various reinforcing fillers, carbon black (CB) has long been the industry standard owing to its excellent dispersion in rubber matrices and its ability to significantly enhance tensile strength, abrasion resistance, and elasticity [3,4,5,6]. In particular, CB-filled elastomers based on ethylene–propylene–diene monomer (EPDM) and polybutadiene rubber (PB) are commonly employed in applications demanding durability and resilience [7].

Blending EPDM and PB serves to synergistically combine the advantageous characteristics of both materials. EPDM is a saturated, non-polar rubber renowned for its superior resistance to heat, ozone, and weathering, making it suitable for long-term outdoor use [8,9,10]. However, its relatively high viscosity and limited unsaturation hinder its curing efficiency [11]. In contrast, PB is an unsaturated diene rubber with low glass transition temperature (T_g_), excellent flexibility, and superior dynamic properties such as high rebound resilience and elasticity [12]. The incorporation of PB into EPDM improves vulcanization efficiency, crosslink density, and processability, while simultaneously introducing flexibility and enhancing energy dissipation [13]. Consequently, EPDM/PB blends provide a balanced elastomeric matrix with tailored mechanical and elastic characteristics, ideal for studying filler interactions and property optimization.

Despite the advantages of CB, its widespread use raises significant environmental concerns. CB is derived from fossil fuels and is non-biodegradable, contributing to persistent solid waste issues, particularly in the case of end-of-life tires [5,14]. Its role in environmental pollution and human health hazards has prompted increasing regulatory and scientific attention [15,16]. Therefore, developing eco-friendly alternatives or partial substitutes for CB is both a technological and environmental imperative [17,18,19].

Mica, a naturally occurring layered silicate mineral, offers a promising alternative due to its abundance, chemical stability, and environmentally benign nature [20,21]. Mica exhibits high aspect ratio, thermal resistance, and inherent rigidity, making it an attractive candidate for enhancing dimensional stability and heat resistance in polymer systems [20,22,23]. Furthermore, mica entails lower environmental impact during its production process compared to carbon black and presents a cost advantage due to its relatively lower price [24,25]. However, the hydrophilic surface and poor interfacial compatibility of mica with non-polar rubbers like EPDM and PB often limit its reinforcement capability [22,26]. To address this challenge, surface modification techniques such as silane coupling have been explored [27,28], and various substances have been used to modify the surface of mica, leading to improvements in dispersibility, tensile strength, modulus, and other functional properties [29,30]. In particular, ureidopropyltrimethoxysilane (URE) is a versatile coupling agent that improves the interfacial adhesion between inorganic fillers and organic polymers by introducing functional groups that facilitate chemical or physical bonding [31]. When the surface of inorganic fillers is modified using URE, the overall mechanical properties of rubber are improved, and excellent reinforcement properties can be observed in terms of tensile strength. In addition, it can contribute to improved dispersion by destroying the agglomerates of inorganic fillers [32].

When URE with these advantages is used to modify the surface of mica, the surface characteristics of mica can be changed to improve interaction with rubber, thereby achieving overall reinforcement of mechanical properties.

In this study, we investigate the effects of incorporating both unmodified and URE-modified mica into EPDM/PB blends, either alone or in combination with CB. A comprehensive evaluation of curing characteristics, mechanical strength, elastic recovery, and dynamic properties is performed to assess the potential of surface-treated mica as a partial CB replacement. The primary objective is to reduce CB usage while maintaining or enhancing the overall performance of the rubber composite, thereby improving the material’s environmental profile without compromising its functional attributes. This work aims to provide a practical route toward more sustainable elastomeric materials by leveraging the synergy between tailored rubber blends and hybrid filler systems.

## 2. Experimental Section

### 2.1. Materials

Ethylene–propylene–diene monomer (EPDM; KEP 330, specific gravity: 0.86) from Kumho Polychem Co. (Seoul, Republic of Korea) and polybutadiene rubber (PB; KBR-01, specific gravity: 0.91), commonly known as butadiene rubber (BR), from Kumho Petrochemical Co. (Seoul, Republic of Korea), were used as the rubber matrix. The EPDM used in this study contains 57 wt% ethylene, 35 wt% propylene, and 8 wt% of a non-conjugated diene, specifically an ethylidene norbornene (ENB) group. Carbon black (CB; FEF) from TSR Co. (Gumi, Republic of Korea) and mica (KT-100) from Kolortek Co., Ltd. (Huaian, Jiangsu, China) were used as fillers. The mica used in this study is synthetic mica. The particle sizes of CB and mica were approximately 40–48 nm and 25 µm, respectively. Ureidopropyltrimethoxysilane (URE), used for surface modification of mica, was purchased from BNOChem Co. (Cheongju, Republic of Korea). Stearic acid (SA), zinc oxide (ZnO), sulfur, tetramethylthiuram disulfide (TMTD), and 2,2-dibenzothiazole disulfide (DM) from Puyang Willing Chemical Co. (Puyang, China) were used as additives. Toluene was purchased from BNO Chem Co. (Cheongju, Republic of Korea). Carbon black, mica, and URE-treated mica are denoted as CB, M, and SM, respectively, in this study.

### 2.2. Rubber Compounding

In the rubber compounding process referred to as the carbon master batch (CMB), the matrix consisted of a rubber blend composed of 90 wt% EPDM and 10 wt% PB. As fillers, a total of 30 phr of CB, M, and SM were added. ZnO and stearic acid were incorporated at fixed contents of 5 phr and 2 phr, respectively (Table 1). ZnO and SA were gradually incorporated into the matrix, after which the fillers were progressively added over a period of 50 min. The compound was then cooled at room temperature for about 30 min, re-masticated, and subjected to the final master batch (FMB) process. In the subsequent FMB process, the vulcanization accelerators; TMTD (1 phr) and DM (0.6 phr) were added, along with sulfur as the curing agent at 1.5 phr. They are sequentially added over a 15 min mixing period.

### 2.3. Modification of Mica

Surface treatment of mica was carried out as follows. First, the pH of 400 mL of distilled water was adjusted to 3.0 using acetic acid. Subsequently, 1 wt% of URE was gradually added, and the mixture was stirred for 2 h to induce hydrolysis. Afterward, 200 g of mica was gradually added and mixed for an additional 30 min. Once mixing was completed, the treated mica was dried at room temperature for 48 h, followed by an additional drying step in an oven for approximately 30 min.

### 2.4. Specimen Preparation

For both the tensile and compression set (CS) specimens, the processing time was determined based on rheological data, and the processing temperature was set to 160 °C. The mold was placed on the press plates, the rubber sample was loaded into the mold, and then, heat and pressure were applied for the predetermined curing time. In the case of CS specimens, an additional 30 s of curing time was applied to ensure complete vulcanization throughout the thicker cross-section, as incomplete curing might occur inside due to the increased specimen thickness.

### 2.5. Characterization

#### 2.5.1. Curing and Rheological Properties

##### Rheometer

To evaluate the rheological properties of the rubber, a rubber rheometer (DRM-100, Deakyung Engineering Co., Bucheon, Republic of Korea) was used. The test was conducted at 160 °C for 30 min. The parameters measured included the time to reach 90% of its maximum torque during curing (T_90_), scorch time (T_s2_), curing rate index (CRI), and delta torque (ΔM). Each result was obtained by averaging measurements from three samples.

##### Mooney Viscosity

To measure the Mooney viscosity of the rubber, a Mooney viscometer (DWV-200C, Deakyung Engineering Co., Bucheon city, Republic of Korea) was used. The test was conducted at 125 °C with a 1 min preheating period followed by a 4-min measurement, totaling 5 min. Each result was obtained by averaging the measurements from three samples.

#### 2.5.2. Crosslink Density

To determine the crosslink density of the rubber, both a swelling experiment and the Flory–Rehner relation were employed. Each specimen (ca. 1 mg) was swollen in toluene for 24 h, and the crosslink density was evaluated by comparing the weights before and after swelling. The results were calculated as the average of three specimens. The crosslink density based on the Flory–Rehner relation was calculated according to the following equation.

$\nu_{e}$ is defined for a perfect network as the number of elastically active network chains per unit volume and is given by

(1) $$\nu_{e}=\rho_{p}N_{A}/M_{c}$$

where *ν_r_* is the volume fraction of swollen rubber and given by following relation:

(2) $$\nu_{r}=1/(1+Q)\;\;$$

where *Q* is defined as grams of solvent per gram of rubber hydrocarbon and calculated by

(3) $$Q=(W-W_{d})/{\;W}_{d}\;\;\;\;\;$$

where *W* is the weight of the swelling test sample during 24 h, ${\;W}_{d}$ is the weight of the sample after 24 h swelling test was ended.

The $\rho_{p}$ is specific gravity of polymer calculated according to equation

(4) $$\rho_{p}=\frac{W_{a}}{{W_{a}-W}_{s}}*\;\rho_{specific\;gravity\;of\;water}\;\;$$

The average molecular weight of the polymer between cross-links ($M_{c}$) according to Flory–Rehner relation is as follows:

(5) $$M_{c}=\rho_{p}\nu_{s}v_{r}^{\frac{1}{3}}/-\ln\left(1-\nu_{r}\right)\;+\;\nu_{r}\;+\;\;{\chi v}_{r}^{2}\;\;$$

The *χ* is the Flory–Huggins interaction parameter, which can be determined by

(6) $$\chi ≅0.34+\nu_{s}{\left(\delta_{s}-\delta_{r}\right)}^{2}/RT\;\;\;$$

where $\delta_{s}$ is the solubility parameter of solvent, $\delta_{r}$ solubility parameter of polymer, *R* is the gas constant (i.e., 8.3144598 J/mol) and *T* (the room temperature).

#### 2.5.3. Mechanical Properties

##### Tensile Properties

The mechanical properties of the rubber were measured using a universal testing machine (UTM; DUT-500CM, Daekyung Engineering Co., Bucheon, Republic of Korea) in accordance with ISO 37 [33]. The tensile test was conducted at room temperature with a crosshead speed of 500 mm/min using a 5 kN load cell. Dumbbell-shaped specimens with a cross-sectional area of 15 mm × 4 mm and a gauge length of 40 mm were prepared for the test. Each result was calculated as the average of five specimens.

##### Hardness

The Shore A hardness was measured using a hardness tester (306 L, Pacific Transducer Instruments, Los Angeles, CA, USA) in accordance with ISO 48-2 [34]. The results were obtained by measuring five specimens and calculating the average.

#### 2.5.4. Elastic Properties

##### Rebound Resilience

The rebound resilience of the rubber was measured using a ball rebound tester (H090, UTS International Co., Zhangzhou, China) in accordance with ISO 4662 [35]. A ball was dropped onto each sample, and the rebound height was measured to calculate the rebound resilience. The results for each sample were obtained by measuring five specimens and calculating the average.

##### Compression Set

The compression set (CS) was measured in accordance with ISO 815 [36]. Cylindrical specimens were prepared and placed in a cylindrical mold with dimensions of 12.5 mm (± 0.5 mm) × 9 mm, then compressed at 125 °C for 22 h. After compression, the specimens were allowed to recover at room temperature for 30 min, and the change in height was measured and recorded. The results for each sample were obtained by measuring four specimens and calculating the average.

##### Tan δ

To evaluate the Tan δ values of the rubber, dynamic mechanical analysis (DMA, DMA850, TA Instruments, New Castle, DE, USA) was performed. The experiment was conducted in compression mode according to ISO 6721-12 [37], using specimens with a diameter of 12.5 mm and a thickness of 2 mm. Measurements were carried out under isothermal conditions at 25 °C with a frequency of 1 Hz, and a single specimen was tested for each measurement.

#### 2.5.5. Morphological Properties

To examine the surface characteristics of EPDM/BR blends, EPDM/BR/CB, EPDM/BR/mica, and EPDM/BR/CB/mica composites, a scanning electron microscope (SEM; Apro, FEI Co., Hillsboro, OR, USA) was used with the mode of energy dispersive X-ray spectrometer (EDS). Si element was detected for EDS mapping. The surface of the rubber samples was observed using the fracture surface of tensile test specimens, and SEM images were taken at an accelerating voltage of 10.0 kV. Prior to imaging, each sample was coated with a 5–10 nm layer of gold particles for 3 min using a sputter coater (Cressington 108 Auto Sputter Coater, Ted Pella Inc., Redding, CA, USA).

## 3. Results and Discussions

### 3.1. Surface Modification of Mica

**Figure 1:**
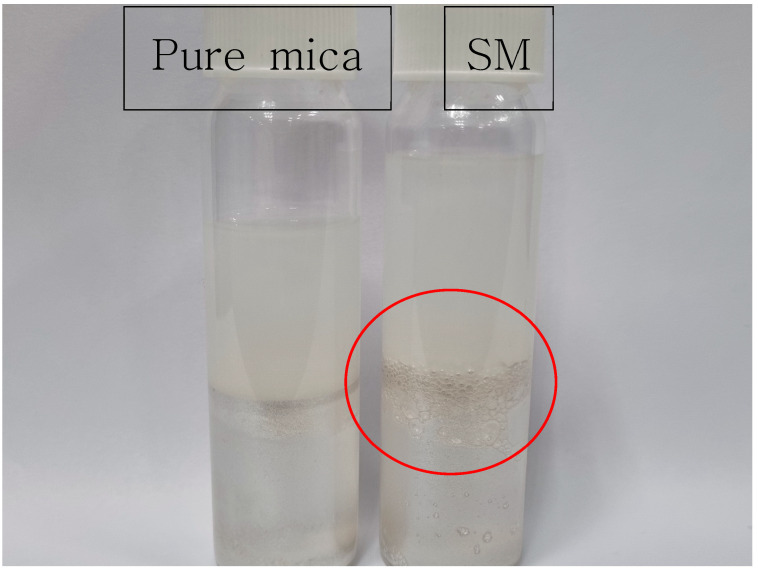
Photographs of Pickering emulsion. The red circle highlights the Pickering emulsion formed by surface-modified mica (SM).

To verify the surface modification of mica, DIW and the hydrophobic substance chloroform were placed in a vial, and mica and SM were, respectively, introduced into the interface formed by the two liquids to form a Pickering emulsion [38]. The corresponding photographs are provided in Figure 1.

Upon examination of the photographs, bubbles can be observed inside the vial containing SM. This phenomenon is attributed to the formation of a hydrophilic coating on the mica surface induced by silane, indicating that the surface modification of the mica was successfully achieved.

### 3.2. Morphological Properties

**Figure 2:**
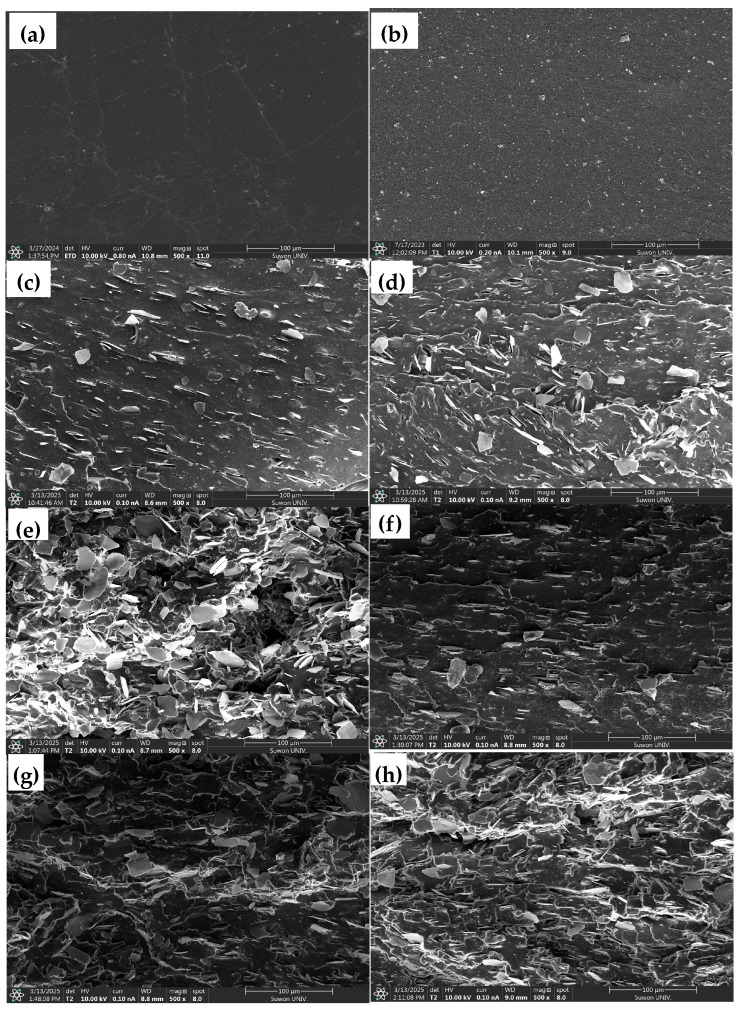
SEM images of fractured surface of rubber blend and EPDM/PB/CB/mica composites: (**a**) E9B1, (**b**) E9B1/CB30, (**c**) E9B1/CB20/M10, (**d**) E9B1/CB10/M20, (**e**) E9B1/M30, (**f**) E9B1/CB20/SM10, (**g**) E9B1/CB10/SM20, and (**h**) E9B1/SM30.

The fractured surfaces of specimens from tensile tests were observed via SEM (Figure 2) and SEM-EDS (Figure S1), and the results are presented in Figure 2 and Figure S1. As shown in Figure 2f–h, which correspond to composites containing surface-modified mica (SM), the interface between the mica particles and the rubber matrix appears to be improved compared to unmodified mica systems shown in Figure 2c–e. Specifically, void formation between filler and matrix was reduced in SM-containing samples, indicating enhanced interfacial adhesion. This observation supports the hypothesis that the URE treatment successfully introduced functional groups on the mica surface that promote stronger interactions with the rubber phase.

Furthermore, the degree of agglomeration among mica particles was lower in the SM-containing composites [23]. In contrast, the unmodified mica systems exhibited larger aggregates and poor filler dispersion, which likely contributed to stress concentration points and deteriorated mechanical performance, as also reflected in the reduced tensile strength, which will be discussed in mechanical studies [39]. The improved dispersion of SM fillers is consistent with the enhanced toughness and tensile modulus, especially in the CB20/SM10 sample, which exhibited the best balance of elasticity and mechanical reinforcement. The sample E9B1/SM30 (Figure 2h) showed relatively uniform filler distribution even at high SM content, unlike the unmodified mica samples, where increased surface-treated filler content led to more pronounced agglomeration. This confirms that surface treatment not only enhances filler–matrix adhesion but also suppresses the tendency for particle clustering at higher loadings.

These SEM findings are further corroborated by the crosslink density results, where surface-modified mica composites exhibited higher crosslink density than their unmodified counterparts. The enhanced network density suggests more effective filler–rubber interaction, leading to improved stress transfer during mechanical deformation. SEM analysis demonstrates that surface modification of mica improves both interfacial compatibility and filler dispersion, which may in turn contribute to enhanced mechanical properties and elastic recovery of the EPDM/PB composites. These morphological improvements reinforce the viability of using surface-treated mica as a partial eco-friendly substitute for carbon black in high-performance rubber applications.

### 3.3. Curing and Rheological Properties

**Figure 3:**
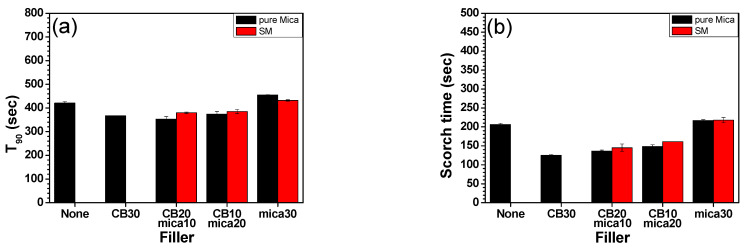

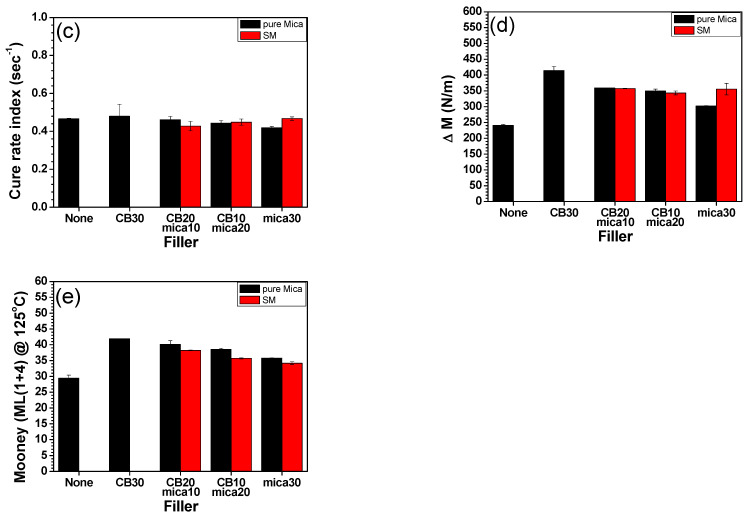
Curing and rheological properties of each rubber and EPDM/PB/CB composites: (**a**) T_90_, (**b**) scorch time (Ts2), (**c**) curing rate index (CRI), (**d**) ΔM, and (**e**) Mooney viscosity.

The rheometer measurements are presented in Figure 3a–d, showing the curing behavior of various EPDM/PB composites. The parameters examined include optimum cure time (T_90_), scorch time (T_s2_), cure rate index (CRI), and delta torque (ΔM). Notably, the T_90_ and T_s2_ values were the highest for the sample containing 30 phr mica without CB, regardless of whether the mica was surface modified. This delayed curing behavior is attributable to the poor thermal conductivity of mica compared to CB. The ability of CB to enhance heat transfer during vulcanization is absent in CB-free samples, resulting in slower cure kinetics.

The SM systems exhibited a similar trend to the unmodified mica (M) systems, indicating that the silane treatment did not significantly influence the bulk heat transfer behavior during curing. This is consistent with the near-identical values of T_90_ and T_s2_ between M and SM samples, especially at equivalent phr loadings (e.g., M20 vs. SM20, and M30 vs. SM30).

The CRI, calculated using Equation (1), showed a decreasing trend with increasing mica content:

(7) $$CRI=\frac{100}{\left(T_{90}-Ts2\right)}$$

This behavior is likely due to the physical adsorption of accelerators onto the high surface area of mica particles, which become more pronounced at higher filler loadings. Such adsorption interferes with the intended function of the accelerators, delaying the onset and progression of crosslinking reactions [40]. This phenomenon is well-known in filler-embedded rubber composite systems with active or polar surfaces, especially in silicate-based fillers [41,42]. Delta torque (ΔM), which reflects the extent of crosslinking and overall stiffness after vulcanization, also followed a similar pattern to T_90_ and CRI. Although no dramatic differences were observed between M and SM systems, a slight decrease in ΔM was noted in the SM30 sample, potentially indicating minor overlubrication or premature filler–filler networking that may reduce torque development. However, the overall similarity across samples suggests that the rubber systems, regardless of mica type or content, reached comparable levels of vulcanization.

Mooney viscosity results (Figure 3e) demonstrated a gradual decline as the mica content increased, which aligns with expectations for layered, plate-like fillers. The incorporation of mica, particularly in high aspect ratio and well-dispersed form, can facilitate matrix flow by acting as a processing aid, thereby reducing internal friction during mixing [27]. This effect was consistent in both M and SM systems, indicating that the silane surface treatment did not significantly alter processability at the compounding stage.

Overall, these findings demonstrate that while mica, especially when used in high proportions without CB, can delay curing (higher T_90_ and T_s2_), it does not compromise the overall curing completeness (as shown by ΔM). Moreover, the addition of surface-modified mica maintains comparable curing behavior while contributing to improved filler–matrix interaction, as evidenced in subsequent mechanical and morphological analyses.

### 3.4. Crosslink Density

**Figure 4:**
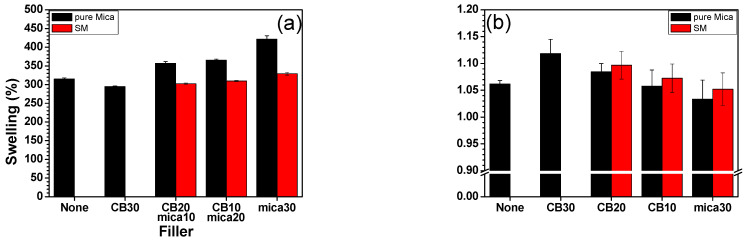
Swelling tests of EPDM/BR/CB/mica rubber composites: (**a**) swelling index and (**b**) crosslink density.

Swelling tests were conducted to evaluate the crosslink density of EPDM/PB composites containing various amounts and types of mica fillers. The results, as illustrated in Figure 4, demonstrate a distinct relationship between swelling behavior, delta torque (ΔM), and the crosslink network structure of the vulcanizates.

Swelling behavior is inversely correlated with crosslink density; a more tightly crosslinked rubber network will resist solvent penetration, resulting in a lower degree of swelling. The parameter Q and *ν_r_* were derived using Equations (3) and (4). These values were then incorporated into the Flory–Rehner equation (Equation (6)) to calculate the *Mc* and the corresponding crosslink density (*ν_e_*) via Equation (2). The data reveal that the degree of swelling increases as a function of mica concentration, indicating a reduction in crosslink density, as shown in Figure 4a,b. This trend is particularly evident in the CB10/M20 and M30 samples. This is caused by two factors, such as physical dilution of the rubber matrix (high loadings of inorganic mica particles reduce the available rubber volume for crosslinking) and adsorption of additives for curing (mica’s polar layered structure can adsorb sulfur and accelerators, thereby limiting their availability during vulcanization, as discussed earlier).

**Figure 5:**
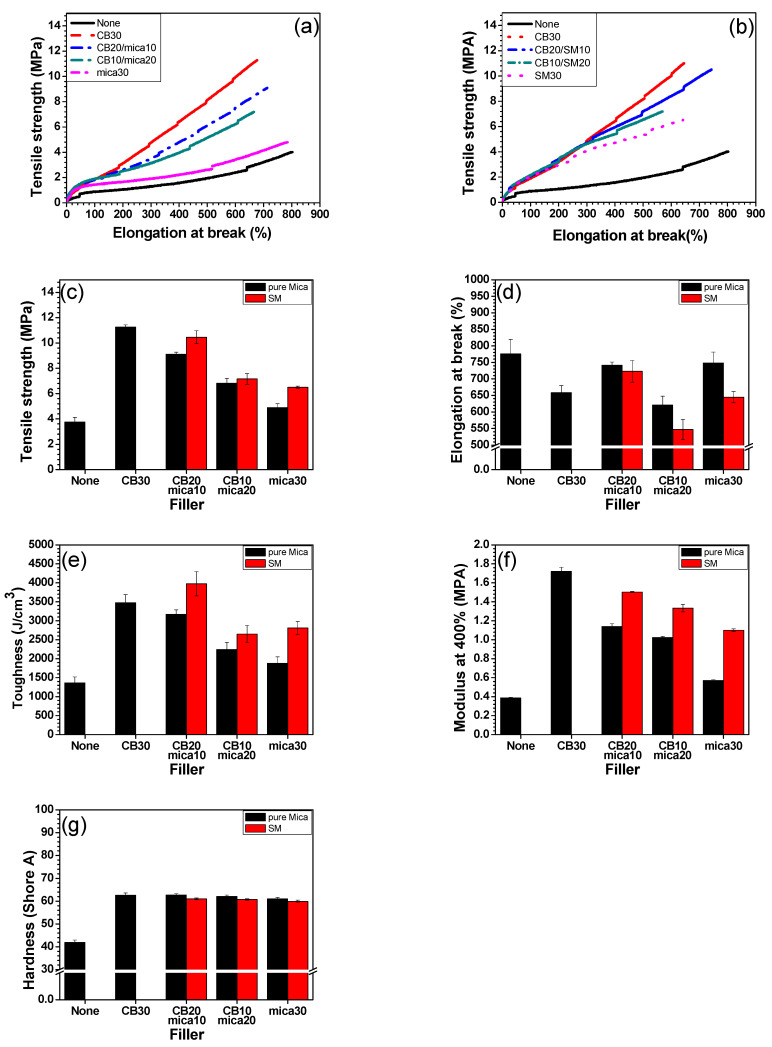
Mechanical properties of EPDM/BR/CB/mica composites: (**a**) SS-curve of CB/mica composites, (**b**) SS-curve of CB/URE composites, (**c**) tensile strength, (**d**) elongation at break, (**e**) toughness, (**f**) modulus at 400%, and (**g**) hardness.

**Figure 6:**
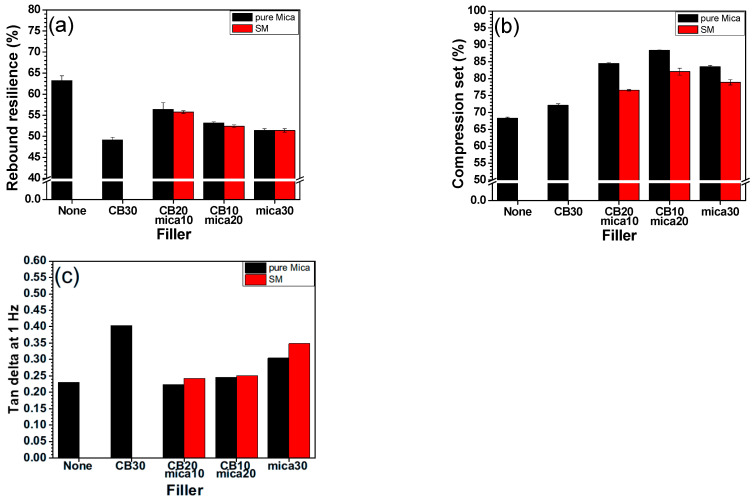
Elastic properties of EPDM/BR/CB/mica rubber composites: (**a**) rebound resilience; (**b**) compression set; (**c**) Tan δ of EPDM/BR/CB/mica rubber composites.

However, composites containing SM, particularly CB20/SM10 and CB10/SM20, show a recovery or even enhancement in crosslink density compared to their unmodified counterparts. This improvement can be attributed to the presence of URE on the mica surface, which introduces functional groups capable of forming stronger chemical or physical interactions with the rubber matrix. These interactions increased the interfacial compatibility, promoting more efficient stress transfer and curing. Moreover, this enhanced interfacial bonding in the SM systems is consistent with the elevated ΔM values observed in rheometer data (Figure 3d) and the superior mechanical properties reported in Figure 5. It also correlates with the higher resilience and better compression set performance shown in Figure 6, suggesting that network integrity improves. The swelling and crosslink density data validate the role of surface-modified mica in reinforcing the rubber network. While unmodified mica tends to disrupt crosslinking efficiency at higher loadings, URE-modified mica not only mitigates this effect but also facilitates a denser, more resilient crosslinked structure [43]. This indicates the effectiveness of surface modification in enabling mica to function as a more compatible and reinforcing filler in eco-friendly rubber composites.

### 3.5. Mechanical Properties

The mechanical properties of the EPDM/PB composites incorporating CB, mica (M), and SM were investigated using uniaxial tensile tests, and the results are summarized in Figure 5. As shown in Figure 5c, the tensile strength generally decreased with increasing content of unmodified mica. This trend is expected, as mica lacks strong interfacial interaction with the rubber matrix due to its inert, hydrophilic nature [44]. In contrast, CB enhanced the tensile strength effectively via strong filler–matrix interaction and the ability to promote strain-induced crystallization. Consequently, composites with CB only (e.g., CB30) exhibited the highest tensile strength. However, when mica was surface modified with URE, an improvement in tensile strength was observed across all filler loadings. In particular, the CB20/SM10 and CB10/SM20 formulations showed significantly higher tensile strength than their unmodified counterparts (CB20/M10 and CB10/M20). This result suggests that URE treatment improved filler dispersion and interfacial adhesion, enabling more efficient stress transfer between the matrix and the mica particles [45]. The elongation at break results in Figure 5d exhibited less consistent trends compared to tensile strength. Nevertheless, SM-containing composites generally maintained higher elongation than those with unmodified mica at equivalent concentration levels. Notably, CB20/SM10 showed a nearly 100% increase in elongation compared to the CB30 sample, which is significant given the slight decrease in tensile strength. This finding implies that the SM filler not only maintained the network flexibility but also alleviated the brittleness typically introduced by high filler content. Consequently, the toughness, which is the area under the stress–strain curve and a combined function of tensile strength and elongation, displayed a non-linear but synergistic trend (Figure 5e). The CB20/SM10 formulation showed the highest toughness among all tested systems, even exceeding that of the CB30 control. This synergistic enhancement is attributed to the balanced reinforcement and ductility conferred by the hybrid CB/SM system. The improved interfacial adhesion from silane treatment likely contributed to this by preventing stress concentration and early crack initiation. The modulus at 400% strain (Figure 5f) showed a trend similar to that of tensile strength, further confirming that reinforcement efficiency was enhanced by URE treatment. While unmodified mica tends to reduce modulus due to its poor interfacial compatibility, SM-loaded systems retained or even improved modulus values, indicating better load transfer and elastic recovery at high deformation. Finally, hardness measurements (Figure 5g) revealed relatively minor changes across all samples. This suggests that Shore A hardness is less sensitive to filler–matrix interfacial quality and is instead more influenced by the bulk filler volume and crosslink density, which remained comparable among the composites.

These mechanical results collectively indicate that surface modification of mica via URE is highly effective in enhancing mechanical reinforcement without compromising ductility. The CB20/SM10 composition emerged as the optimal formulation, achieving a superior combination of strength, elongation at break, and toughness. This emphasizes the potential of surface-treated mica as a sustainable and functional co-filler for partial replacement of CB in high-performance elastomeric materials.

### 3.6. Elastic Properties

The elastic performance of the EPDM/PB composites filled with CB, M, and SM was evaluated using rebound resilience and compression set (CS) tests, and the results are illustrated in Figure 6. The rebound resilience results (Figure 6a) exhibited a non-linear trend with increasing mica content. Specifically, the incorporation of 10 phr mica led to an initial increase in rebound resilience for both M and SM systems. However, as mica content further increased (to 20 and 30 phr), the resilience gradually declined, regardless of whether the mica was surface modified. This behavior can be explained by the hysteresis mechanism in rubber: energy applied during deformation is partly stored elastically and partly dissipated as heat due to internal friction between molecular chains. The initial increase in resilience with low mica content suggests that well-dispersed mica flakes reduce internal chain friction, thereby lowering hysteresis losses and increasing the elastic energy return [46,47]. This may be attributed to lamellar slippage or reduced entanglement density near mica surfaces [48].

The increasing presence of surface-treated particles leads to reduced polymer mobility, greater filler–filler interactions, and poorer dispersion. This results in higher internal friction and hysteresis, ultimately reducing rebound elasticity. Moreover, as seen in Figure 4, the decrease in crosslink density at high mica loading further enhanced the molecular mobility, contributing to increased energy dissipation. The SM systems showed slightly lower rebound resilience compared to their unmodified counterparts at the same filler loading. This counterintuitive result may stem from premature filler–filler interactions or self-agglomeration of SM particles due to silane bridging, which impedes optimal stress distribution and elastic recovery.

The compression set results (Figure 6b) showed a generally inverse trend to resilience, as expected: lower compression set values indicate better elastic recovery after prolonged compressive strain and are desirable for applications requiring long-term dimensional stability. Among the tested samples, the CB10/M20 formulation exhibited a lower volume loss (i.e., better CS performance) compared to CB20/M10, despite the higher total mica content. This suggests that, at an optimal CB-to-mica ratio, the composite benefits from both thermally stable filler characteristics (from mica) and sufficient network elasticity (from CB). Since mica is an inherently heat-resistant material, its incorporation enhanced the thermal dimensional stability, which plays a more significant role in compression set (contributable to the condition of elevated temperatures) than in rebound resilience. In addition, SM-containing samples exhibited similar or marginally improved CS values over M-containing ones, implying that silane-modified interfaces help preserve elastic structure under compressive loads.

The incorporation of mica, especially at moderate levels, had a positive impact on the elastic performance of EPDM/PB composites. While rebound resilience benefited from low mica loadings owing to reduced chain friction, higher loadings compromised elasticity unless counterbalanced by improved interfacial adhesion through surface modification. Compression set performance reflects a more consistent enhancement, attributable to the thermal stability of mica and improved interfacial bonding in SM systems. The CB20/SM10 and CB10/SM20 composites emerge as possibly promising formulations that balance elasticity, recovery, and thermal durability.

The Tan δ values of the rubber blend and EPDM/PB/CB/mica rubber composites were evaluated using DMA at 1 Hz frequency under isothermal conditions, as shown in Figure 6. Tan δ is defined as the ratio of the loss modulus (*E*″) to the storage modulus (*E*′), as shown below:

(8) $$Tan\delta =\frac{E^{\prime \prime }}{E^{\prime }}$$

Here, E′ (storage modulus) represents the elastic response of the material, i.e., the energy stored and recovered during deformation, while E″ (loss modulus) indicates the viscous response, i.e., the energy dissipated as heat. Therefore, Tan δ serves as a direct indicator of viscoelastic damping and hysteresis behavior in rubber. A higher Tan δ value corresponds to greater energy loss and lower elastic recovery, implying increased hysteresis. Conversely, a lower Tan δ value suggests that the material stores more energy elastically, reflecting better elasticity. This interpretation is consistent with the rebound resilience results shown in Figure 6a, which exhibit an inverse relationship with Tan δ. Samples with lower Tan δ demonstrated higher rebound resilience, confirming the validity of Tan δ as a complementary metric for evaluating the elastic behavior of rubber composites. On the other hand, the correlation between Tan δ and compression set (Figure 6b) was less direct, since compression set also reflects thermal resistance in addition to elastic recovery. Overall, the Tan δ results support the conclusion that low filler content, especially when using surface-modified mica, improved the elastic energy return of the composite by reducing internal friction and damping.

The Tan δ values increased with increasing mica content up to 30 phr in both M and SM systems. Composites containing 10 phr mica (e.g., CB20/M10 or CB20/SM10) exhibited the lowest Tan δ, indicating minimal energy loss and hence higher elasticity. In contrast, samples with higher mica loadings (20–30 phr) displayed elevated Tan δ, reflecting greater internal damping due to filler–matrix incompatibility or filler agglomeration. This trend confirms that small amounts of mica, particularly when well-dispersed, reduced the internal friction by inhibiting molecular chain entanglement and thereby enhanced the elastic energy return. These findings align with the rebound resilience results (Figure 6a), where an inverse relationship with Tan δ was observed. Samples with high Tan δ values exhibited low rebound resilience, indicating that more energy was dissipated rather than recovered elastically.

While the Tan δ and rebound resilience graphs show mirrored trends, the correlation between Tan δ and compression set (CS) is less straightforward. Compression set depends on both elastic recovery and thermal stability under static load, whereas Tan δ is measured under dynamic, oscillatory conditions. For instance, in the CB10/M20 system, the compression set was lower (indicating good recovery), but the Tan δ value was relatively high. This discrepancy likely arises because mica, being thermally stable, helps resist permanent deformation under compressive stress despite increased damping. Although the differences in Tan δ between M and SM systems were subtle, SM-embedded composites generally exhibited slightly lower Tan δ values than unmodified ones at equivalent concentration levels. This implies that the improved interfacial compatibility due to silane treatment helped reduce energy loss, the stress transfer and network homogeneity was enhanced, and overall improvement in dynamic elasticity was achieved. In addition, the T_g_ values were obtained using DSC and DMA as a function of temperature at a fixed frequency (1 Hz). The T_g_ values rarely changed as shown in Figure S2.

DMA-derived Tan δ values provided valuable insights into the viscoelastic nature of rubber composites. The data validate that low filler content, particularly of surface-treated mica, optimized the energy storage and elastic recovery. Moreover, the Tan δ serves as a complementary metric to rebound resilience, offering a mechanistic understanding of hysteresis and internal damping. The observed inverse relationship with rebound resilience, and partial correlation with compression set, reinforces the role of filler–matrix interactions in dictating dynamic and static elasticity in EPDM/PB-based systems.

## 4. Conclusions

This study demonstrated the potential of mica, SM, and the associated hybrid filler systems (CB/SM) as partial eco-friendly alternatives to CB in EPDM/PB composites. While the incorporation of unmodified mica reduced the crosslink density and mechanical performance owing to poor interfacial adhesion, surface modification using URE improved the filler dispersion and matrix compatibility. As a result, the incorporation of SM into the rubber blend enhanced the tensile strength, toughness, and dynamic elasticity compared to their unmodified counterparts. Among the tested formulations, the hybrid filler system containing 20 phr CB and 10 phr SM (CB20/SM10) achieved the most balanced performance across mechanical, elastic, and dynamic properties, even outperforming the CB-only reference in toughness and elongation. Morphological analysis confirmed the improved interfacial adhesion and reduced filler agglomeration in SM systems, supporting the observed enhancements in both mechanical reinforcement and energy dissipation behavior. Through this study, comprehensive property evaluation studies for the CB/mica hybrid filler system were obtained, and furthermore, balanced improvements in composite performance were achieved by modifying the surface of mica using URE and using the hybrid filler systems. These findings suggest that surface-modified mica can serve as a sustainable co-filler in rubber composites, reducing dependence on CB while maintaining or even improving composite performance. This work provides a practical approach to developing environmentally responsible elastomeric materials for automotive and industrial applications, and paves the way for further research into advanced hybrid filler systems.

## Figures and Tables

**Table 1:** Recipes of the rubber compounding.

Rubber Matrix (wt%)								
PB	10	10	10	10	10	10	10	10
EPDM	90	90	90	90	90	90	90	90
**Additive (phr)**								
Zinc oxide	5	5	5	5	5	5	5	5
stearic acid	2	2	2	2	2	2	2	2
CB	-	30	20	10	-	20	10	-
Mica (M)	-	-	10	20	30			
URE-Mica (SM)						10	20	30
TMTD	1	1	1	1	1	1	1	1
DM	0.6	0.6	0.6	0.6	0.6	0.6	0.6	0.6
Sulfur	1.5	1.5	1.5	1.5	1.5	1.5	1.5	1.5

## Data Availability

The original contributions presented in this study are included in the article/Supplementary Material. Further inquiries can be directed to the corresponding author(s).

## Supplementary Materials

The following supporting information can be downloaded at: https://www.mdpi.com/article/10.3390/polym17162250/s1, Figure S1. SEM-EDS mapping images of fractured surface of rubber blend and EPDM/PB/CB/mica composites: (a) E9B1/CB20/M10, (b) E9B1/CB10/M20, (c) E9B1/M30, (d) E9B1/CB20/SM10, (e) E9B1/CB10/SM20, and (f) E9B1/SM30; Figure S2. Glass transition temperatures of composites: (a) DSC and (b) DMA; Table S1. Glass Transition Temperature in DMA and DSC.
