# Supplementary material for: Effect of Surface-Modified Mica in Hybrid Filler Systems on the Curing and Mechanical Behavior of Ethylene–Propylene–Diene Monomer (EPDM)/Butadiene Rubber (BR) Blend
Source: Polymers (Basel). 2025 Aug 20;17(16):2250. doi: 10.3390/polym17162250 (PMC12389428; doi:10.3390/polym17162250)
Supplement: Supplementary file 1 [file polymers-17-02250-s001.zip › polymers-3758874-supplementary.pdf]

Effect of Surface-Modified Mica in Hybrid Filler Systems on the Curing and Mechanical Behavior of Ethylene–Propylene–Diene Monomer (EPDM)/Butadiene Rubber (BR) Blend

Won-Young Jung †, Seong-Woo Cho † and Keon-Soo Jang *

Department of Materials Science and Engineering, School of Chemical and Materials Engineering,
The University of Suwon, Hwaseong 18323, Gyeonggi-do, Republic of Korea;
wyjung0220@naver.com (W.-Y.J.); 519jo@naver.com (S.-W.C.)
* Correspondence: ksjang@suwon.ac.kr
† These authors contributed equally to this work.

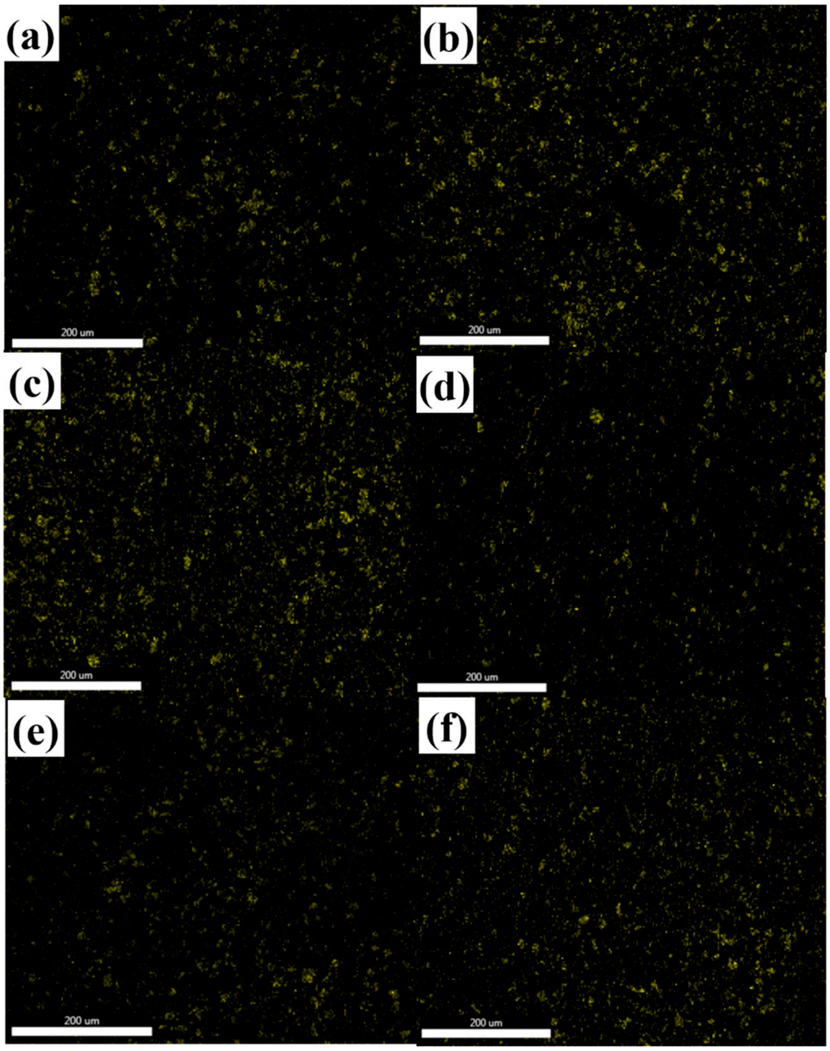

Figure S1. SEM-EDS mapping images of fractured surface of rubber blend and EPDM/PB/CB/mica composites: (a) E9B1/CB20/M10, (b) E9B1/CB10/M20, (c) E9B1/M30, (d) E9B1/CB20/SM10, (e) E9B1/CB10/SM20 and (f) E9B1/SM30.

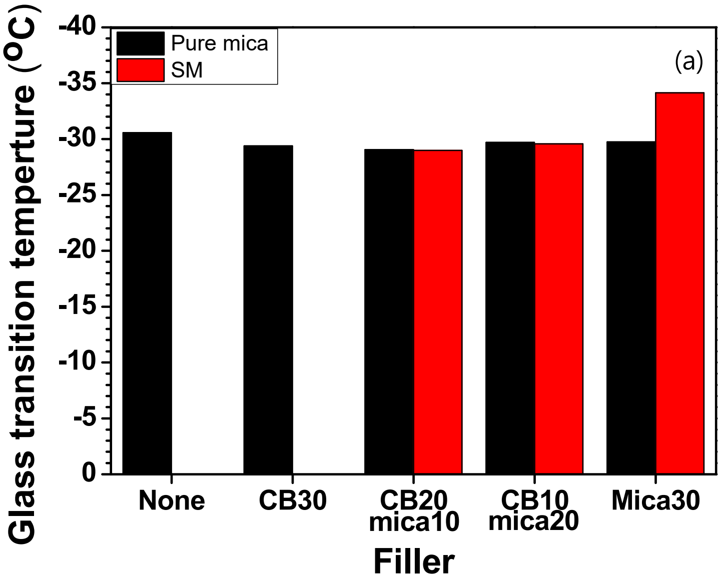

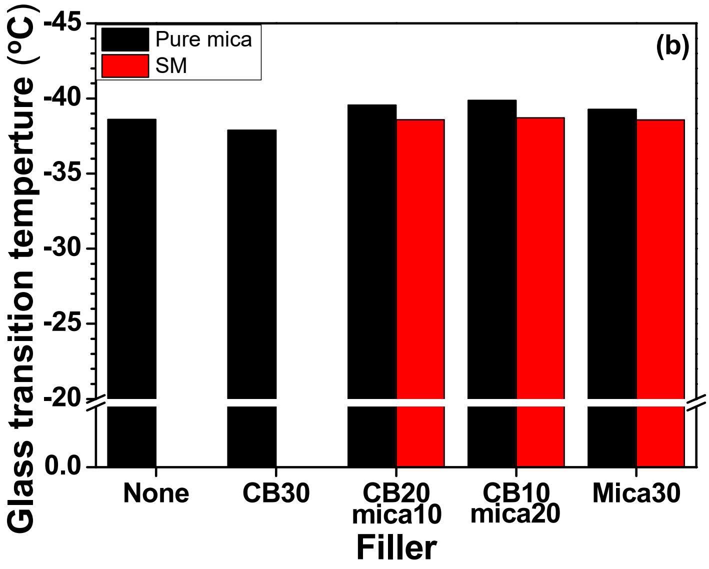

Figure S2. Glass transition temperatures of composites: (a) DSC and (b) DMA

Table S1. Glass Transition Temperature in DMA and DSC

	Filler	None	CB30	CB20/mica10	CB20/mica10	Mica30
Tg in DMA(°C)	Pure mica	-38.6	-38.21	-39.55	-39.86	-39.27
	SM			-38.59	38.7	-38.57
Tg in DSC(°C)	Pure mica	-30.58	-29.38	-29.05	-29.7	-29.741
	SM			-28.98	-29.55	-34.14
